# Associations between infant and young child feeding (IYCF) practice and attitudes toward intimate partner violence (IPV) in Timor-Leste

**DOI:** 10.1186/s12905-023-02206-5

**Published:** 2023-02-13

**Authors:** Kyoko Sasaki, Mika Watanabe, Leonard Ximenes, Cipriano Pacheco, Michiyo Higuchi

**Affiliations:** 1 Nagoya City University Graduate School of Nursing, Nagoya, Japan; 2 Faculty of Public Health, Universidade da Paz, Dili, Timor-Leste

**Keywords:** Infant and young child feeding, Intimate partner violence, Women, Empowerment, Maternal health, Timor-Leste

## Abstract

**Background:**

Both the proportions of malnutrition among children and women’s justifying partner’s intimate partner violence (IPV) are high in Timor-Leste. However, no study has looked at the associations between acceptable infant and young child feeding (IYCF) and women’s attitudes toward IPV, as a women’s empowerment index. In light of the lack of evidence described above, the study objective was to examine associations between IYCF practice and attitudes toward IPV in Timor-Leste and other women’s characteristics.

**Methods:**

A secondary analysis of children’s records from the Demographic and Health Survey Timor-Leste 2016 was conducted using a cross-sectional design. Univariable and multivariable analyses were performed to investigate associations between acceptable IYCF and women’s agreement that men are justified in beating their wives for five specific reasons and socio-economic factors.

**Results:**

The day before the survey, 33.4% of mothers gave their child at least the minimum dietary diversity and 46.4% at least the minimum meal frequency; and 15.0% practiced acceptable IYCF. Among all respondents, 17.5% did not agree that all five specific reasons for beating are justified. The adjusted odds ratio (aOR) of acceptable IYCF for mothers who did not agree was 1.51 (95% confidence interval [CI] 1.09–2.09) compared to those who agreed. The aOR of acceptable IYCF for mothers who worked outside the home was 1.48 (95% CI 1.16–1.96) compared to those who did not. Compared to mothers in the poorest quintile, the aORs of acceptable IYCF among those in the poorer, middle, richer, and richest quintiles were 1.33 (95% CI 0.83–2.21), 1.90 (95% CI 1.15–3.14), 2.01 (95% CI 1.17–3,45), and 2.99 (95% CI 1.63–5.50) respectively. Compared to children aged 6–11 months, the aORs of acceptable IYCF for children aged 12–17 months and 18–23 months were 2.14 (95% CI 1.54–2.97) and 1.63 (95% CI 1.14–2.34), respectively.

**Conclusions:**

Acceptable IYCF was associated with mothers’ attitudes toward wife-beating, wealth quintile, and mother’s work. Factors that we found associated with IYCF were all related to women’s empowerment. It implies that women’s empowerment is a key to improving mothers’ childcare. Merely promoting a specific childcare practice may not be enough for better child health.

## Background

The Convention on the Rights of the Child (United Nations Human Rights, 2002) guarantees that the rights to life, survival and development include medical, educational and livelihood support to protect the lives of all children and to fully develop and grow their abilities. Under the Convention on the Rights of the Child, the World Health Organization (WHO) [1] states that all infants have the right to adequate nutrition. However, undernutrition has been shown to be associated with 45% of child mortality. In 2020, it was estimated that 149 million children under the age of five were stunted, 45 million were underweight and 38.9 million were obese worldwide [1].

Around the age of six months, exclusive breastfeeding no longer meets an infant’s nutritionary requirements and complementary foods are necessary. In addition, WHO states that optimal nutrition for the period up to two years of age is effective for better development of the infant [1]. Further, not exclusively breastfeeding in the first six months of life, introducing baby food too early, inadequate quality and quantity of complementary food, and late start of adequate infant and young child feeding (IYCF) significantly raise the risk of malnutrition [2, 3]. However, in many developing countries less than a quarter of infants aged 6–23 months meet the criteria of dietary diversity and feeding frequency that are appropriate for their age [2].

Timor-Leste is one such country. Although the prevalence of stunting among children under five years old declined from 58% in the 2010 Timor-Leste Demographic and Health Survey (TLDHS) to 46% in 2016, about half of the children are still stunted, which is high compared with the rest of the world. Regarding children in Timor-Leste who received appropriate IYCF, the rate is below the average of 25% for low- and middle-income countries [4].

Several social factors result in poor implementation of appropriate IYCF, one of which is women’s empowerment [5]. Women’s experience of intimate partner violence (IPV), which is a major global health issue, particularly in low- and middle-income countries, is often used as a negative indicator of women’s empowerment [6]. IPV affects women’s physical and mental health through direct pathways, such as injury, and indirect pathways, such as chronic health problems that arise from prolonged stress. An association has also been revealed between IPV experienced by women and the and adverse social and health effects for their children [7]. Previous studies indicated that mothers exposed to any form of IPV (physical, sexual, or emotional violence) were less likely to initiate breastfeeding early, breastfeed exclusively [6] and to practice acceptable IYCF [8]. In addition to IPV, attitudes toward partner’s IPV behavior are often used as an indicator of women’s empowerment [9, 10]. However, few previous studies have investigated the associations between IYCF and attitudes toward IPV.

In Timor-Leste, 47% of ever-married women experienced IPV [11]. In addition, more than four-fifths of women aged 15–49 agreed with at least one question justifying IPV [12]. IYCF, a known factor associated with child nutrition, is associated with IPV, and can be a proxy-indicator of women’s empowerment. In this study, we hypothesize that women’s attitudes to agree that IPV is justified, even not experiencing actual IPV, is associated with poor IYCF. As this is still underreported, findings from a country where both malnutrition and IPV are social issues can contribute to filling the research gap. In light of the lack of evidence described above, the study objective was to examine associations between IYCF practice and attitudes toward IPV in Timor-Leste.

## Methods

### Used data

The study was based on secondary data analysis using data from the TLDHS in 2016. The Demographic and Health Survey (DHS) has been conducted in more than 90 developing countries using representative sampling, for which ICF International, with financial support from the United States Agency for International Development, provides technical support (https://www.dhsprogram.com/data/Using-DataSets-for-Analysis.cfm). TLDHS 2016 was the third DHS in Timor-Leste and was a nationally representative cross-sectional survey implemented between September and December of 2016 by the General Directorate of Statistics under the Ministry of Planning and Finance. The Ministry of Health also collaborated with the implementation of the survey. For TLDHS 2016, a stratified cluster random sampling technique was applied for the selection of households for data collection. The 2015 Timor-Leste Population and Housing Census was used as the sampling frame for the TLDHS. The DHS was approved by the ethics committee of ICF International Inc. and the Ministry of Health of Timor-Leste. An informed consent statement, given to all participants, emphasizes that participation is voluntary; that the respondent may refuse to answer any question or terminate participation at any time. The statement also confirms that the respondent's identity and information will be kept strictly confidential (https://dhsprogram.com/Methodology/Protecting-the-Privacy-of-DHS-Survey-Respondents.cfm). Following analysis, the data were de-identified and made available for public use. We requested data online from the website www.dhsprogram.com and obtained all seven TLDHS 2016 datasets (Women, Households, Men, Children, All Births, Household Members, Couples).

### Data extraction

We used the Children dataset which comprised information on 7221 children under five years of age. For the present study, data from the subgroup of mothers whose child was (1) aged 6–23 months and (2) living with the survey respondent (mother) were extracted. In order to avoid the selection of multiple children from the same mother, information about the mother’s youngest child was used if the mother had more than one eligible children. Finally, data from 1854 mother and child pairs were extracted for this study.

### Variables

#### Minimum acceptable IYCF

**Fig. 1:**
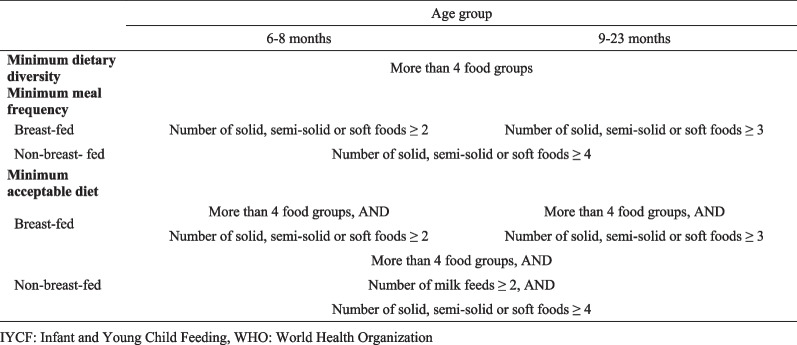
Definition of IYCF indicators by WHO guideline. *IYCF* Infant and Young Child Feeding, *WHO* World Health Organization

Minimum acceptable IYCF was used as the outcome variable in this study. Three core IYCF indicators for appropriate complementary feeding were assessed to describe adherence to the WHO guidelines: minimum dietary diversity, minimum meal frequency and minimum acceptable diet (Fig. 1). Minimum dietary diversity is defined as feeding the child food from at least four of the standard seven food groups. The seven groups comprise: grains, roots, and tubers; legumes and nuts; dairy products (milk yogurt, cheese); flesh foods (meat, fish, poultry, and liver/organ meat); eggs; vitamin A-rich fruits and vegetables; and other fruits and vegetables. By consuming food from at least four food groups, the child has a high likelihood of consuming at least one animal source of food and at least one fruit or vegetable, in addition to a staple food such as grains, roots, or tubers [4]. Minimum meal frequency is necessary for a child to reach their energy requirements [4]. For infants and young children, the indicator is based on how much energy the child needs. Exclusively breastfeeding after six months is unlikely to meet the energy requirements of the child. Breastfed children are considered to be consuming minimum meal frequency if they receive solid or semi-solid foods at least twice a day for children aged 6–8 months and at least three times a day for children aged 9–23 months. Minimum meal frequency for non-breastfed children aged 6–23 months is considered to be adequate if the child receives solid or semi-solid foods at least four times a day. Minimum acceptable diet, a combination of the previous two indicators, is defined differently for breastfed and non-breastfed children and for different age groups [4]. The detailed criteria are shown in Fig. 1.

#### Attitudes toward IPV

“Attitudes toward wife-beating” was used as the main explanatory variable of analyses in the study (Although the term ‘wife beating’ was used in the original questionnaire, since its use is now rare, we refer to ‘intimate partner violence [IPV] in this study). In the DHS model questionnaire, respondents were asked if they agreed that a man is justified in beating his wife in some situations (https://dhsprogram.com/data/Guide-to-DHS-Statistics/Attitude_towards_Wife_Beating.htm).

In TLDHS 2016, respondents were asked about five scenarios in which a husband would be justified in beating his wife: she goes out without telling him, she neglects the children, she argues with him, she refuses to have sex with him and she burns the food. A ‘don’t know’ answer was counted as an ‘agree’ in this study as the participant did not actively disagree that the man was justified in beating his wife.

#### Other explanatory variables

Mothers’ and children’s characteristics were used as co-variables for this study. Maternal age was recoded into seven groups: 15–19, 20–24, 25–29, 30–34, 35–40, 41–44, 45–49 years. Maternal education level was classified as: no education, primary and incomplete primary, incomplete secondary, and secondary and higher. Mother’s work was re-categorized as: not working outside the home (not working, housewife and household work) and working outside. Residential location was defined as either rural or urban. Wealth index was based on the DHS composite measure of a household’s ownership of selected assets, which were already categorized into quintiles of all the households that participated in DHS 2016: poorest, second, middle, fourth and wealthiest [4]. Maternal exposure to mass media (newspaper, radio, television) was divided into three categories: not at all, less than once a week and at least once a week. Frequency of antennal care (ANC) visits was categorized as: 0, 1–3 and ≥ 4 times during the pregnancy. Sex of the child was categorized as male or female. Age of the child was recoded into three groups: 6–11, 12–17 and 18–23 months old.

### Data analysis

First, descriptive statistics were calculated for the variables used. Second, univariable analyses were performed to investigate associations between acceptable IYCF and women’s agreement that a husband is justified in using physical IPV against his wife. Associations between acceptable IYCF and mothers’ and children’s characteristics were also examined. Third, to estimate effect size, odds ratios (ORs) of acceptable IYCF and their 95% confidence intervals (CIs), a logistic regression was performed. Crude ORs (cORs) for attitudes toward IPV, maternal age, maternal education level, mother’s work, residential location, wealth quintile, maternal access to media, frequency of ANC, sex of the child, and age of the child were separately calculated. The combined indicator of attitudes toward IPV (agree with at least one scenario or disagree with all) and all participant characteristics, except exposure to newspaper/magazine and radio, were included in the model and mutually adjusted to calculate adjusted ORs (aORs) and 95% CI. Exposure to television was selected among the media exposure indicators as it was the most commonly reported. Stata/SE version 12.1 (Stata Corp) was used for all statistical analyses. Statistical significance was set at P < 0.05.

## Results

### Participant characteristics

Table 1 shows study participant characteristics of the extracted 1854 respondents. Respondents aged 25–29 years accounted for the highest proportion (30.4%), with a small minority aged 45–49 years (1.8%). More than 60% of the mothers had reached more than primary education (66.8%). The majority of women did not work outside the home (71.0%). The majority of women resided in rural areas (73.0%). Mothers reported access to information through reading newspapers or magazines at least once per week (4.6%), listening to the radio (13.1%) and watching television (32.6%). The majority of women had visited ANC four or more times during their pregnancy with the youngest child (76.3%). Regarding the children, 51.4% were male and 48.6% were female; 35.0% were aged 6–11 months, 37.7% were aged 12–17 months and 27.3% were aged 18–23 months.

**Table 1:** Participant characteristics (N = 1854)

Variables	n	%
*Age*		
15–19	76	4.1
20–24	415	22.4
25–29	564	30.4
30–34	458	24.7
35–39	196	10.6
40–44	112	6.0
45–49	33	1.8
*Education level*		
No education	408	22.0
Incomplete primary	207	11.2
Primary and incomplete secondary	595	32.1
Secondary and higher	644	34.7
*Mother's work*		
No work and home	1318	71
Work outside the home	535	28.9
Don't know	1	0.1
*Area of residence*		
Urban	500	27.0
Rural	1354	73.0
*Wealth index*		
Poorest	362	19.5
Second	397	21.4
Middle	395	21.3
Fourth	419	22.6
Wealthiest	281	15.2
*Exposure to media*		
Newspaper or magazine		
Not at all	1518	81.9
Less than once per week	251	13.5
At least once per week	85	4.6
Radio		
Not at all	1194	64.4
Less than once per week	418	22.6
At least once per week	242	13.1
Television		
Not at all	859	46.3
Less than once per week	391	21.1
At least once per week	604	32.6
*Number of ANC visits*		
Not visit	244	13.2
1–3	195	10.5
4-	1415	76.3
*Child's sex*		
Male	953	51.4
Female	901	48.6
*Child's age of months*		
6–11	649	35.0
12–17	699	37.7
18–23	506	27.3

*ANC* Antenatal care

### Acceptable IYCF

Table 2 shows the results of minimum dietary diversity, minimum meal frequency, and acceptable IYCF practice. Approximately one third of mothers gave their child at least the minimum dietary diversity and less than half of mothers provided at least the minimum meal frequency. Only 15% of all mothers could provide minimum acceptable IYCF.

**Table 2:** Results of acceptable IYCF practice (N = 1854)

	Diversity		Frequency		Acceptable	
	n	%	n	%	n	%
Breast-fed 6–8 months (n = 311)			150	48.2	25	8.0
Breast-fed 9–23 months (n = 910)			465	51.1	177	19.5
Non-breast-fed 6–23 months (n = 633)			245	38.7	76	12.0
All	619	33.4	860	46.4	278	15.0

*IYCF* Infant and Young Child Feeding

### Attitudes toward IPV

Table 3 shows attitudes toward IPV. The percentages of women who disagreed that a man is justified in using physical violence against his wife in the following scenarios were as follows; 26.6% for “she goes out without telling her husband”, 28.5% for “she neglects the children”, 31.6% for “she argues with husband”, 52.0% for “she refuses to have sex with husband” and 60.2% for she burns the food. Only 17.5% of women disagreed with all scenarios.

**Table 3:** Attitudes toward IPV (N = 1854)

Attitudes toward IPV	n	%
*Goes out without telling husband*		
Agree	1361	73.4
Disagree	493	26.6
*Neglects the children*		
Agree	1326	71.5
Disagree	528	28.5
*Argues with husband*		
Agree	1269	68.5
Disagree	585	31.6
*Refuses to have sex with husband*		
Agree	890	48
Disagree	964	52
*Burn the food*		
Agree	738	39.8
Disagree	1116	60.2
*All circumstances*		
Agree with at least one	1530	82.5
Disagree with all	324	17.5

*IPV* Intimate Partner Violence

### Associations between acceptable IYCF and attitudes toward IPV

Acceptable IYCF showed significant associations with attitudes toward IPV for “she argues with husband” and “she burns the food” in the univariable analyses (Table 4). Table 4 shows the results of the logistic regression tests, with the cORs of acceptable IYCF for each variable and aORs which were mutually adjusted. Maternal age, maternal education level, residential location, exposure to media and ANC visits had significant associations in the univariable analysis, but these were not found in the multivariable analysis. Mothers who disagreed that a husband’s IPV in all circumstances was justified were more likely to do acceptable IYCF than those who agreed with at least one (aOR 1.51, 95% CI 1.09–2.09). Mother’s working outside the home was similarly associated with acceptable IYCF significantly (aOR 1.48, 95% CI 1.12–1.96). The richer household the mother lived in, the more likely the mother did IYCF. Compared to mothers in the poorest quintile, the aORs of acceptable IYCF among those in the second, middle, fourth, and wealthiest quintiles were 1.33 (95% CI 0.83–2.21), 1.90 (95% CI 1.15–3.14), 2.01 (95% CI 1.17–3,45), and 2.99 (95% CI 1.63–5.50) respectively. Children aged 12–17 months were more likely to receive acceptable IYCF than children aged 6–11 months (aOR 2.14, 95% CI 1.54–2.97), which was slightly decreased among children aged 18–23 months (aOR 1.63, 95% CI 1.14–2.34) (Table 4).

**Table 4:** Results of univariable odds ratio for predictors of acceptable IYCF practice, Timor-Leste (N = 1854)

Variables	n	%	cOR	95% CI	*p *value	aOR	95% CI	*p *value
*Attitudes toward IPV*								
Goes out without telling husband								
Agree	1361	73.4						
Disagree	493	26.6	1.31	0.99–1.73	0.06			
Neglects the children								
Agree	1326	71.5						
Disagree	528	28.5	1.22	0.96–1.61	0.16			
Argues with husband								
Agree	1269	68.5						
Disagree	585	31.6	1.45	1.10–1.87	0.007			
Refuses to have sex with husband								
Agree	890	48.0						
Disagree	964	52.0	1.12	0.86–1.44	0.40			
Burn the food								
Agree	738	39.8						
Disagree	1116	60.2	1.61	1.22–2.11	0.001			
All circumstances								
Agree with at least one	1530	82.5						
Disagree with all	324	17.5	1.51	1.11–2.06	0.009	1.51	1.09–2.09	0.01
Age								
15–19	76	4.1						
20–24	415	22.4	1.82	0.75–4.39	0.18	1.49	0.61–3.64	0.39
25–29	564	30.4	1.93	0.81–4.59	0.14	1.38	0.57–3.35	0.48
30–34	458	24.7	2.39	1.00–5.71	0.05	1.68	0.69–4.09	0.26
35–39	196	10.6	2.28	0.91–5.69	0.08	1.51	0.59–3.88	0.39
40–44	112	6.0	2.38	0.91–6.28	0.08	1.70	0.63–4.59	0.30
45–49	33	1.8	3.14	0.97–10.21	0.06	2.30	0.68–7.75	0.18
Education level								
No education	408	22.0						
Incomplete primary	207	11.2	0.77	0.44–1.32	0.34	0.81	0.46–1.43	0.47
Primary and incomplete secondary	595	32.1	1.16	0.80–1.69	0.44	1.06	0.71–1.60	0.77
Secondary and higher	644	34.7	1.72	1.21–2.46	0.003	1.21	0.79–1.85	0.38
Mother's work								
No work and home	1319	71.1						
Work outside the home	535	28.9	1.59	1.22–2.07	0.001	1.48	1.12–1.96	0.007
Area of residence								
Urban	500	27.0						
Rural	1354	73.0	0.61	0.46–0.79	< 0.001	0.98	0.70–1.37	0.89
Wealth index								
Poorest	362	19.5						
Second	397	21.4	1.39	0.85–2.29	0.19	1.33	0.83–2.21	0.27
Middle	395	21.3	2.10	1.29–3.29	0.003	1.9	1.15–3.14	0.01
Fourth	419	22.6	2.46	1.56–3.88	< 0.001	2.01	1.17–3.45	0.01
Wealthiest	281	15.2	3.96	2.49–6.29	< 0.001	2.99	1.63–5.50	< 0.001
*Exposure to media*								
Newspaper or magazine								
Not at all	1518	81.9						
Less than once per week	251	13.5	1.47	1.03–2.08	0.03			
At least once per week	85	4.6	2.65	1.63–4.33	< 0.001			
Radio								
Not at all	1194	64.4						
Less than once per week	418	22.6	1.35	0.99–1.83	0.06			
At least once per week	242	13.1	1.88	1.33–2.66	< 0.001			
Television								
Not at all	859	46.3						
Less than once per week	391	21.1	1.24	0.88–1.75	0.23	0.81	0.55–1.20	0.29
At least once per week	604	32.6	1.74	1.31–2.33	< 0.001	0.98	0.68–1.43	0.93
Number of ANC visits								
Not visit	244	13.2						
1–3	195	10.5	1.41	0.78–2.54	0.25	1.25	0.68–2.30	0.47
4–	1415	76.3	1.76	1.13–2.75	0.01	1.37	0.86–2.18	0.19
Child's sex								
Male	953	51.4						
Female	901	48.6	1.11	0.86–1.43	0.44	1.10	0.85–1.43	0.48
Child's age of months								
6–11	649	35.0						
12–17	699	37.7	2.21	1.60–3.04	< 0.001	2.14	1.54–2.97	< 0.001
18–23	506	27.3	1.77	1.25–2.52	0.001	1.63	1.14–2.34	0.01

*aOR* adjusted Odds Ratio, *ANC* Antenatal care, *cOR* crude Odds Ratio, *CI* Confidence Interval, *IPV* Intimate Partner Violence, *IYCF* Infant and Young Child Feeding

## Discussion

Our current study using a nationally representative dataset showed that mothers who disagreed that a husband is justified in using IPV were more likely to practice IYCF than those who agreed, while previous studies have suggested exposure to IPV was associated with poor IYCF. We also found that acceptable IYCF were significantly associated with wealth quintile, mother’s work, and child’s age.

It has been reported that agreeing with the justification for IPV perpetrated by their husbands is one of the factors that puts women in a vulnerable environment [13]. According to another report, mothers who have been exposed to IPV have social issues, such as lack of family support, restricted access to services, strained relationships with healthcare providers and employers, and isolation from social networks [5]. Mother’s autonomy in childcare is important for good child health outcomes [14–16], so the isolation of mothers may adversely affect their independence. Such social issues are considered to be related to women’s empowerment, and women with low empowerment were less likely to practice acceptable IYCF [5]. Therefore, empowering women can improve a variety of health indicators [17]. In this study, negative attitudes toward IPV as part of empowerment may have been positively related to IYCF as one such health indicator.

Our study showed that women from higher wealth quintiles were more likely to practice acceptable IYCF than those from the lower quintiles and those who worked outside the home were more likely to practice acceptable IYCF than those who did not. Different countries have different factors that influence the implementation of appropriate IYCF [18].Our findings about the associations with wealth index and with mother’s working status were similar to previous studies [19,20,21. If the mother works outside the home, she gives the family greater economic advantages, thus making it possible to access a wider variety of foods, which may lead to better IYCF. In a society where the extended family still plays an important role, the senior woman in the family can undertake childcare and give the child appropriate IYCF, even if the mother works outside the home and spends less time with her child. In Timor-Leste, senior women in the family, such as grandmothers, have a great deal of power to make decisions regarding childcare when the mother works outside [22].

As previously discussed, a mother’s feeling of isolation can reduce her sense of empowerment and her implementation of IYCF [8]. In addition to improving women’s socio-economic status, working outside the home may prevent women from feeling isolated. Furthermore, women who work outside the home can connect with society, and access to information and support from those around them, all of which are believed to increase the implementation of IYCF. Not justifying IPV, household wealth, and working outside might interact with each other and lead to women’s empowerment, which was associated with better IYCF practice. However, we did not examine statistical interactions in this study.

Children aged 12 to 17 months were more likely to be given appropriate IYCF than those aged 6–11 months, but those aged 18 to 23 months showed a lower proportion than 12 to 17 months in Timor-Leste. A previous study in Ethiopia also showed that as children grew up, the IYCF implementation rate decreased [19]. In Timor-Leste, 90% of mothers continue breast feeding when the baby is 6 months old, but less than 40% when the baby is 23 months old [23]. As a mother become more familiar with feeding her baby, she might pay less attention to the quality of feeding [24]. In the case of Timor-Leste, it may be necessary to promote awareness of the need for adequate IYCF, especially for children over 18 months. Mothers should be advised that continued breastfeeding for two years is recommended to enable appropriate IYCF practice with minimum meal frequency [1].

In our study there was no significant difference between the IYCF practice of those who visited ANC less often and those who visited more often. However, a previous study showed that ANC visits were strongly associated with appropriate IYCF in low- and middle-income countries [25]. The proportion of mothers having at least four ANC visits increased from 55% in 2009–2010 to 77% in 2016 in Timor-Leste [26] but simply increasing the number of ANC does not seem to lead to acceptable IYCF implementation. WHO has increased the recommended number of ANC visits from four or more to eight or more [27]. It might therefore be necessary to increase the number of ANC visits, and to improve the content of ANC and the teaching skills of specialists in Timor-Leste.

This study was based on a national representative data and its selection bias is considered to be small. Nevertheless, there are some limitations related to the study design. First, because of the cross-sectional design of the study, causal relationships cannot be clarified. Second, the data were retrospectively collected from the mothers, potentially resulting in recall bias. Third, there is a limit to possible generalization, as mothers were only asked about their IYCF practice the day before the survey.

## Conclusions

The study indicated that acceptable IYCF was associated with mother’s attitudes toward IPV, the mother’s work, and her economic status in Timor-Leste. Findings from a country where both malnutrition and IPV are social issues contribute to filling the research gap. Factors that we found associated with IYCF were all related to women’s empowerment. It implies that women’s empowerment is a key to improving mothers’ childcare. Merely promoting a specific childcare practice may not be enough for better child health.

## Data Availability

The dataset used and analyzed during this study is publicly available from the DHS website at https://dhsprogram.com/Data/

## Abbreviations

ANC: Antenatal Care
aOR: Adjusted Odds Ratio
CI: Confidence Intervals
cOR: Crude Odds Ratio
DHS: Demographic and Health Survey
IPV: Intimate Partner Violence
IYCF: Infant and Young Child feeding
OR: Odds Ratio
TLDHS: Timor-Leste Demographic and Health Survey
WHO: World Health Organization
